# MEDICAL FINANCIAL HARDSHIP AND EMOTIONAL WELL-BEING AMONG US OLDER ADULTS WITH AND WITHOUT ALZHEIMER’S DISEASE

**DOI:** 10.1093/geroni/igae098.3144

**Published:** 2024-12-31

**Authors:** Shinae Choi, Maria Pisu

**Affiliations:** University of Alabama, Tuscaloosa, Alabama, United States; University of Alabama at Birmingham, Birmingham, Alabama, United States

## Abstract

This study investigated the impact of medical financial hardship (MFH), specifically the inability to make medical payments, on various dimensions of emotional well-being (EWB), including positive and negative affect, life satisfaction, and sense of meaning and purpose in life, among U.S. older adults with and without Alzheimer’s disease and related dementias (ADRD). Data were from the 2016-2020 waves of the Health and Retirement Study (N=12,904). Multivariate regression analyses were computed adjusting for health and sociodemographic factors. Overall, 884 respondents faced MFH, and 352 self-reported a physician diagnosis of ADRD. In people without ADRD, 6.7% faced MFH, while among those with ADRD, the percentage rose to 12.2% (χ2(1)=16.324, p< 0.001). In people without ADRD, MFH was significantly associated with lower life satisfaction (b=-1.195, p< 0.001), positive affect (b=-0.301, p< 0.001), sense of meaning and purpose in life (b=-0.417, p< 0.001), and with higher negative affect (b=0.454, p< 0.001). In those with ADRD, MFH was significantly associated with lower life satisfaction (b=-1.193, p< 0.01) and higher negative affect (b=0.661, p< 0.01); associations with positive affect (b=-0.459) and sense of meaning and purpose in life (b=-0.315) were nonsignificant at the p< 0.05 level. Findings suggest that MFH is twice as likely in people with ADRD, potentially impacting several aspects of their EWB, including life satisfaction and negative affect. The impact is broader for older adults without ADRD, where MFH may also affect their evaluation of meaning and purpose in life. We discuss implications for further research and interventions to improve EWB of older adults facing MFH, both with and without ADRD.

